# A call for governments to pause Twitter censorship: using Twitter data as social-spatial sensors of COVID-19/SARS-CoV-2 research diffusion

**DOI:** 10.1007/s11192-020-03843-5

**Published:** 2021-02-28

**Authors:** Vanash M. Patel, Robin Haunschild, Lutz Bornmann, George Garas

**Affiliations:** 1 Department of Surgery and Cancer, Imperial College London, 10th Floor, Queen Elizabeth the Queen Mother Wing, St. Mary’s Hospital, London, W2 1NY UK; 2 Department of Colorectal Surgery, West Hertfordshire NHS Trust, Watford General Hospital, Vicarage Road, Watford, Hertfordshire, WD18 0HB UK; 3 Max Planck Institute for Solid State Research, Heisenbergstraße 1, 70569 Stuttgart, Germany; 4 Division for Science and Innovation Studies, Administrative Headquarters of the Max Planck Society, Hofgartenstr. 8, 80539 Munich, Germany

**Keywords:** Altmetrics, Twitter, Spatial maps, COVID-19, SARS-CoV-2

## Abstract

In this study we determined whether Twitter data can be used as social-spatial sensors to show how research on COVID-19/SARS-CoV-2 diffuses through the population to reach the people that are affected by the disease. We performed a cross-sectional bibliometric analysis between 23rd March and 14th April 2020. Three sources of data were used: (1) deaths per number of population for COVID-19/SARS-CoV-2 retrieved from John Hopkins University and Worldometer, (2) publications related to COVID-19/SARS-CoV-2 retrieved from World Health Organisation COVID-19 database, and (3) tweets of these publications retrieved from Altmetric.com and Twitter. In the analysis, the number of publications used was 1761, and number of tweets used was 751,068. Mapping of worldwide data illustrated that high Twitter activity was related to high numbers of COVID-19/SARS-CoV-2 deaths, with tweets inversely weighted with number of publications. Regression models of worldwide data showed a positive correlation between the national deaths per number of population and tweets when holding number of publications constant (coefficient 0.0285, S.E. 0.0003, *p* < 0.001). Twitter can play a crucial role in the rapid research response during the COVID-19/SARS-CoV-2 pandemic, especially to spread research with prompt public scrutiny. Governments are urged to pause censorship of social media platforms to support the scientific community’s fight against COVID-19/SARS-CoV-2.

## Introduction

Twitter is a social network created in 2006, that brings together hundreds of millions of users around its minimalist concept of microblogging, allowing users to post and interact with messages known as ‘tweets” (https://about.twitter.com/en_us/company.html, 2020). Twitter has short delays in reflecting what its users perceive, and its principle of “following” users without obligatory reciprocity, together with a very open application programming interface, make it an ideal medium for the study of online behaviour (Grandjean 2016). Tweets can be used as ‘social sensors’, which is the concept of transforming a physical sensor in the real world through social media analysis. Tweets can be regarded as sensory information and Twitter users as sensors. Studies have demonstrated that tweets analysed as social sensors can provide insight into major social and physical events like earthquakes (Sakaki et al. 2010), sporting events (Takeichi et al. 2014), celebrity deaths (Sankaranarayanan et al. 2009), and presidential elections (Shamma et al. 2009). Twitter data contain location information which can be converted into geo-coordinates and spatially mapped. In this way, tweets can be used as social-spatial sensors to demonstrate how research diffuses within a population (Bornmann et al. 2020).

Researchers are increasingly using Twitter as a communication platform, and tweets often contain citations to scientific publications (Priem and Costello 2010). Twitter citations can form part of a rapid dialogue between users which may express and transmit academic impact and support traditional citation analysis. Twitter citations are defined as direct or indirect links from a tweet to a peer-reviewed scholarly article online (Priem and Costello 2010; Sakaki et al. 2010), and reflect a broader discussion crossing traditional disciplinary boundaries, as well as representing ‘attention, popularity or visibility’ rather than influence (Mas-Bleda and Thelwall 2016).

Coronavirus disease 2019 (COVID-19) is a novel infectious disease caused by severe acute respiratory syndrome coronavirus 2 (SARS-CoV-2). The World Health Organization (WHO) declared the 2019–2020 coronavirus outbreak a Public Health Emergency of International Concern (PHEIC) [Statement on the second meeting of the International Health Regulations (2005) Emergency Committee regarding the outbreak of novel coronavirus (2019-nCoV) 2020] on 30 January 2020 and a pandemic on 11 March 2020 (Ghebreyesus 2020).

We use Twitter data as social-spatial sensors to demonstrate how research on COVID-19/SARS-CoV-2 diffuses through the population and to investigate whether research reaches the people that are especially affected by the disease. Bornmann et al. (2020) demonstrated using HIV, tuberculosis and malaria as examples that Twitter data can be used for this purpose.

## Summary of related studies

In our previous study, we have summarized previous research on Twitter and spatial analysis of online activities (Bornmann et al. 2020). As well as our previous study, which investigated how Twitter may be used as social-spatial sensors to track research diffusion in countries affected by HIV, tuberculosis and malaria, other studies have also shown how Twitter can be used to monitor public concerns with infectious diseases that cause epidemics and pandemics. Studies have used textual analysis of tweets from live Twitter chats to determine public and institutional concerns about Ebola and Zika diseases (Glowacki et al. 2016; Lazard et al. 2015). More recently, textual analysis of tweets related to COVID-19/SARS-CoV-2 has shown that it is possible to understand the major public concerns including public fears and trending topics of the disease (Leelawat et al. 2020).

## Methods

### Dataset used

We used three sources of data in this study: (1) deaths per number of population for COVID-19/SARS-CoV-2, (2) publications related to COVID-19/SARS-CoV-2, and (3) tweets of these publications. All data was retrieved and analyzed between 23rd March and 14th April 2020. We used this time frame because COVID-19/SARS-CoV-2 had been declared a global pandemic by the WHO. By the end of March, all European countries and more than 150 countries worldwide had been affected by the disease.

### Deaths

We used deaths per number of population as a measure of severity of the outbreak of the virus in countries and USA states. We used deaths per one hundred thousand population for country specific data, which was retrieved from Coronavirus Resource Center at John Hopkins University (Mortality Analyses 2020). We used deaths per one million population for US state specific data, which was retrieved from Worldometer, a provider of global COVID-19 statistics trusted by institutions such as the United Kingdom government and The Center for Systems Science and Engineering at Johns Hopkins University (Worldometer’s COVID-19 data 2020).

### Publications

We used the WHO COVID-19 database of global publications, which is the latest international multilingual scientific findings and knowledge on COVID-19 from searches of bibliographic databases, hand searching, and the addition of other expert-referred scientific articles (Global research on coronavirus disease (COVID-19) 2020). In total, 2413 publications were downloaded in CSV format from the WHO COVID-19 database of global publications on 23 March 2020, out of which 1941 publications had DOI information. Some data cleaning was performed for increasing the number of valid and removing invalid DOIs using R (R Core Team 2019). Strings like “dx.doi.org” and “doi:” before a DOI were removed. In the case of some publications, a DOI was located in the column “Accession Number”. In these cases, the DOI column was empty and the DOI from the column “Accession number” was used.

For some publications, multiple DOIs occurred in the same field delimited with whitespaces. A few manual checks revealed that sometimes one of the DOIs seemed to be related to research on coronavirus disease but others were not. We decided to remove all publications which contained whitespaces in the DOI field. After the cleaning procedure, some DOIs occurred multiple times. The R package plyr was used during the data cleaning process (Wickham 2011). Finally, we obtained a set of 1782 unique DOIs which occurred only once in the dataset. The countries of the author’s affiliations were downloaded via the Dimensions API (see https://www.dimensions.ai/). We found 1761 out of 1782 DOIs. The R packages rjson (Couture-Beil 2014) and sqldf (Grothendieck 2017) were used for parsing and aggregating the response from the Dimensions API.

### Tweets

We used Altmetric.com application programming interface to extract tweet identifiers for any tweets which mentioned any of the publications on Twitter (Bonasio 2014). The tweet IDs of the tweets which mentioned any of the publications on Twitter were downloaded from the Altmetric.com API using R (R Core Team 2019) with the R packages httr (Wickham 2017a) and RCurl (Lang and the CRAN team 2018). The tweets were downloaded between 27 March and 07 April 2020 via the Twitter API using R (R Core Team 2019) and stored in a local SQLite database file using the R package RSQLite (Müller et al. 2017). Functions from the R package DBI were used for sending database queries (R Special Interest Group on Databases (R-SIG-DB) et al. 2018). The R package ggplot2 was used for plotting the time evolution of tweets (Wickham 2016). The R package tidyverse (Wickham 2017b) was used for analysis of the Twitter user profiles. The R package UpSetR (Gehlenborg 2019) was used for plotting classifications of Twitter user profiles.

In total, 757,133 tweets were downloaded which mentioned 1561 DOIs (87.6% of the DOIs extracted from the WHO data set). In the case of 6065 tweets, no tweet text and no meta-data could be retrieved via the Twitter API. We analyzed the remaining 751,068 tweets. Not all Twitter users provide information about their geographical location (Sakaki et al. 2010; Wouters et al. 2019). Only 13 tweets contained precise geo-coordinates but 494,137 of those tweets contained some free-text user location information. We discarded the precise geo-coordinates and used only the user location information. One problem with the free-text user location information is that some users seem to become very imaginative. In order to reduce wrong location information, we needed to filter the location information for meaningful entries. In summary, we imported the city and country names from the Global Research Identifier Database (GRID, https://grid.ac/) for obtaining a whitelist of existing cities and countries and performed data cleaning of the free-text user location information (Bornmann et al. 2020).

The unique location strings were passed to the Google API via the R package ggmap if the location strings contained more than three characters (Kahle and Wickham 2013). The Google API returned among others precise geo-coordinates, country, and state names (if available) which were stored in a CSV file for plotting and statistical analysis. Overall, the geo-coordinates of 118,994 tweets could be determined and used for further analysis.

### Statistical analysis

We used several Stata commands in this study (Crow and Gould 2013; Huebler 2012; StataCorp 2017). The most important Stata commands were shp2dta (Crow 2006) and spmap (Pisati 2007) to produce the Twitter maps. We additionally calculated Poisson regression models with number of tweets as dependent variable and COVID-19/SARS-CoV-2 deaths per number of population and number of publications as independent variables. We included another binary independent variable reflecting national censorship of Twitter in Iran and China (1 = national censorship). With count variables as dependent variables, Poisson regression models are indicated (Deschacht and Engels 2014; Hilbe 2014). We focus on percentage changes in expected counts in the interpretation of the models (Long and Freese 2014). These percentages can be interpreted as follows: for a standard deviation increase in the death rate per population in a country (or US state), the increases in the expected tweet number in that country (or US state), holding the country’s (or US state’s) number of publications constant.

## Results

**Fig. 1:**
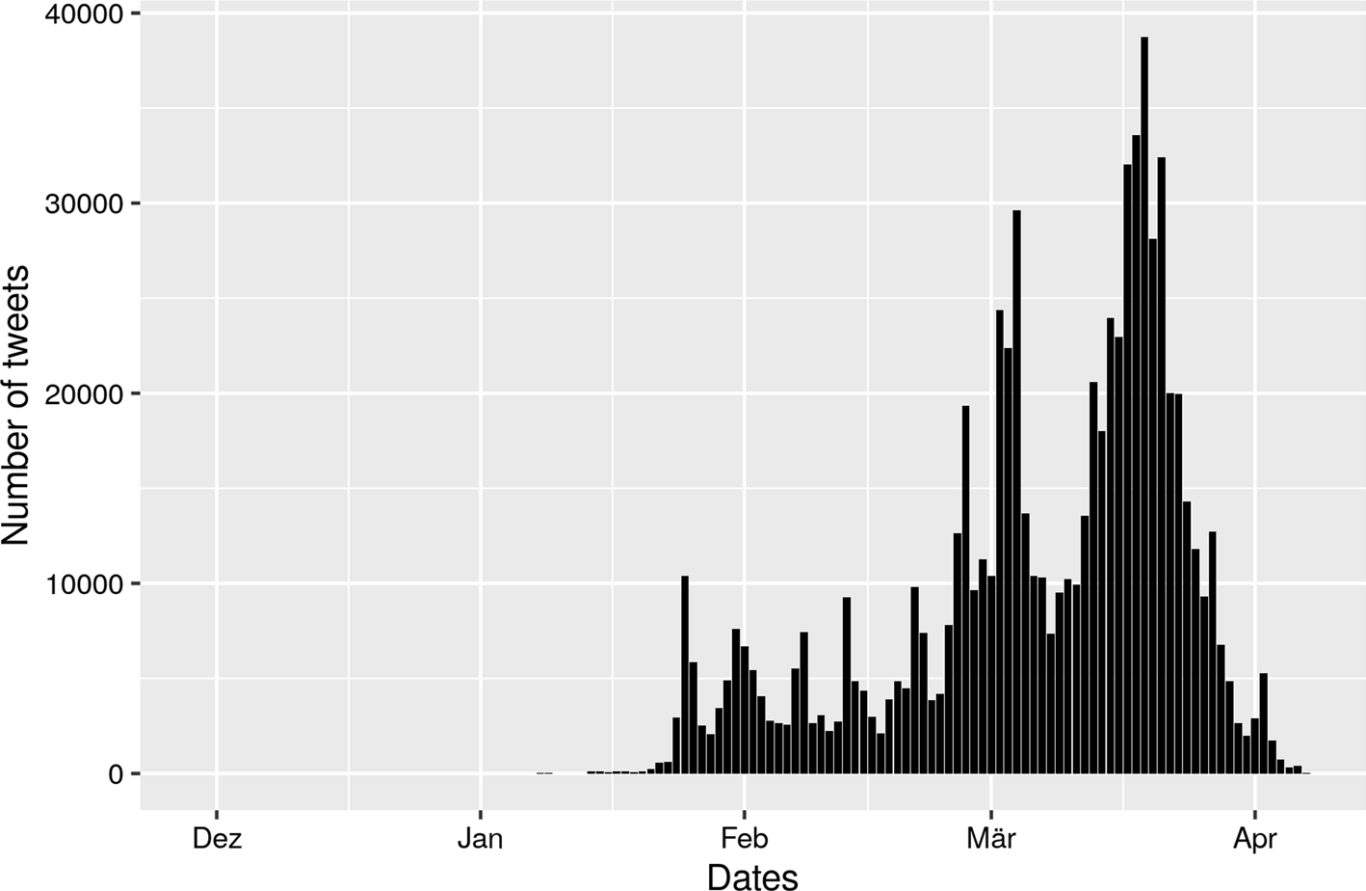
Tweet timeline showing number of tweets per day since 17th November 2019

The deaths per one hundred thousand population for countries ranged from 0 (Ethiopia) to 104 (San Marino). The deaths per one million population for USA states ranged from 2 (Wyoming) to 513 (New York). The total number of publications that were used in the analysis was 1761, and the total number of tweets that were used in the analysis was 751,068. Figure 1, which shows a barplot of the number of tweets per day since 17th November 2019 (date of the first known case of COVID-19/SARS-CoV-2), demonstrates exponential growth in twitter activity in March 2020.

The activity of Twitter sensors—which can be in the status “active” (i.e., tweeting) or not—on certain triggers (e.g., earthquakes or indications of influenza) can be measured. In this study, Twitter users’ function as social-spatial sensors by being aware of publications dealing with a certain disease. Since one can expect that the interest in publications on certain diseases increases, when the user is located in regions with many cases of illness, Twitter rates and disease rates might correlate. This relationship can only be assumed, however, if the general public is active on Twitter (besides researchers) and tweets about scholarly publications. Altmetric.com uses a popular classification system for Twitter users which is not ideal for our study because the analyses cannot be limited to only those Twitter users for whom we have geographical location information, and Altmetric.com defines the group ‘members of the public’ as people who do not tweet links to scholarly publications. In order to receive information on the people tweeting on COVID-19/SARS-CoV-2 research, we used the classification scheme proposed by Toupin et al. (2019) (Table 1) and a modified version of the R code provided by Toupin (2020).

**Table 1:** Twitter user classification scheme proposed by Toupin et al. (2019)

User	Profile
Faculty and students	Higher education or the realm of research
Communicators and journalists	Transmission of information at higher scale (e.g., media, arts, literature)
Professionals	Engaging with research publications relevant to their job (e.g., conservation manager)
Political	Engaging with research publications with political interest (e.g., through activism or as part of governmental jobs)
Personal	Self-describe themselves using personal interests (e.g., in cats or dogs)
Institutions and organizations	Represent a group of people
Bots	Use keywords related to automated activity
Journals and publishers	Represent journals or scientific publishers

**Fig. 2:**
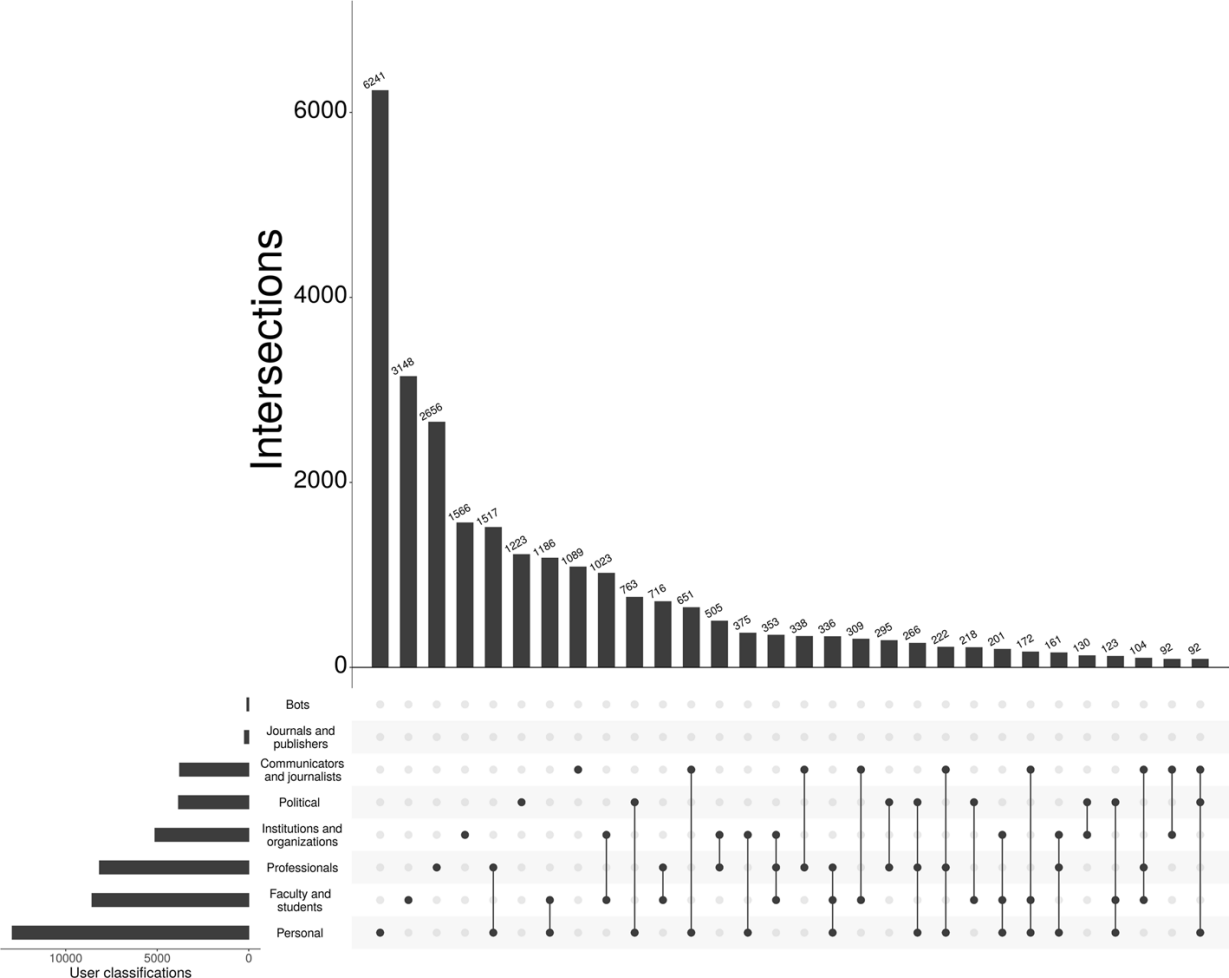
Twitter user classification for tweets on publications dealing with COVID-19/SARS-CoV-2. The graph is restricted to the set of profiles with geographical location information that could be converted into geo-coordinates

The result of the analysis is displayed in Fig. 2, which shows the number of tweets per user classification for tweets on publications dealing with COVID-19/SARS-CoV-2. The visualization reveals that many profiles (and their tweets) are assigned to more than one classification. There seems to be a large proportion of members of the general public in the dataset of this study. The largest proportion of Twitter user profiles belongs to the classification “Personal”. The second largest classification is “Faculty and students”. Other classifications that could be understood as members of the general public, too, (e.g., “Professionals”, “Institutions and organizations”, and “Political”) also contribute with sizable proportions. The classification with the lowest number of profiles is “Bots”.

### Mapping worldwide data

**Fig. 3:**
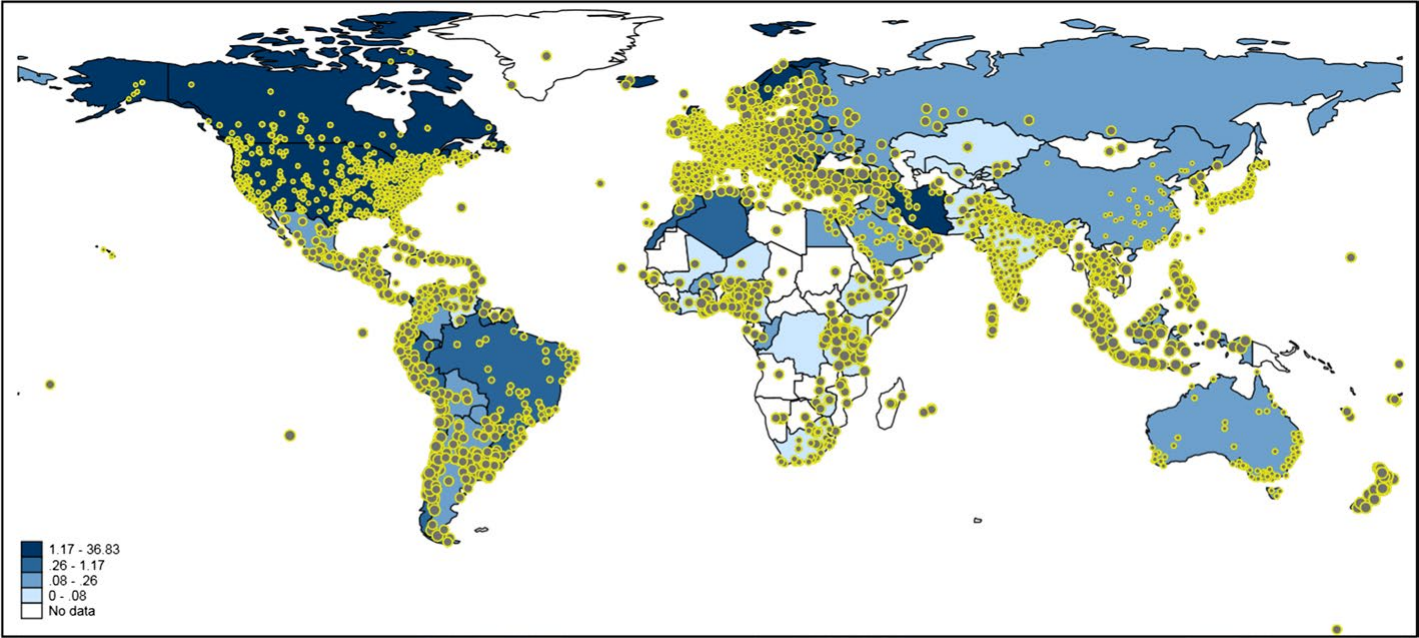
Tweeting on publications dealing with COVID-19/SARS-CoV-2 worldwide. Each tweet is inversely weighted with the number of publications published by authors in the corresponding country: the larger the dots, the smaller the research activity. The countries are colored according to the national deaths per one hundred thousand population. For some countries, e.g. Greenland, no data are available. Countries such as China and Iran block internet access to Twitter or its content (Mortality Analyses 2020)

Figure 3 shows worldwide Twitter activity referring to publications dealing with COVID-19/SARS-CoV-2. The underlying blue-colored scheme visualizes national deaths per number of population. The map is intended to show whether COVID-19/SARS-CoV-2 research reaches regions with many COVID-19/SARS-CoV-2 deaths: does the number of COVID-19/SARS-CoV-2 cases correlate with the number of tweets on COVID-19/SARS-CoV-2 publications?

One of the problems with Twitter data in the context of this study is that Twitter activity is generally high where more research is done (e.g., Western Europe or the Boston area in Fig. 1). Since this is not the activity which we intended to measure, we inversely weighted the size of each tweet on the map by the number of publications in that country [i.e., 1/log(number of publications)]. Thus, the more publications’ authors are located in a country, the smaller the size of the tweet dot is (see here Ginsberg et al. 2009; Sinnenberg et al. 2017). We assume that large dots reflect tweets of people not doing research or not being a publisher/publishing organization (but might be personally confronted with COVID-19/SARS-CoV-2).

The map in Fig. 3 might show the expected result that high Twitter activity is related to high numbers of COVID-19/SARS-CoV-2 deaths. However, it is not completely clear whether this conclusion can be drawn, since there are several countries with high Twitter activity and high publication output (e.g., Western Europe and the Boston region). For some regions on the map, the extent of Twitter activity is difficult to interpret since tweet dots might overlap (especially those with larger sizes). To have a conclusive answer on the relation between Twitter activity and publication output, we additionally calculated Poisson regression models with number of tweets as dependent variable and deaths per number of population and number of publications as independent variables. In order to control the influence of research activity on Twitter activity, the number of publications has been considered as a second independent variable in the model.

The results are shown in Table 2. The coefficients of deaths per number of population and number of publications are statistically significant. The percentage changes in expected counts reveal that deaths per number of population and Twitter activities are related in fact: for a standard deviation increase in the national deaths per number of population, the expected number of tweets in that country increases by 19.7 percentage points, holding the country’s number of publications constant. The results in Table 2 further show that the influence of the number of publications is significantly higher than that of deaths per number of population.

**Table 2:** Coefficients of a Poisson regression model with number of tweets as dependent variable (n = 111 countries)

Independent variable	Coefficient	Standard error	Percentage change in expected count
Deaths per number of population	0.0285***	0.0003	19.7
Number of publications	0.0152***	2.82 × 10^−5^	155.5
National censorship of Twitter	− 8.6662***	0.0376	
Constant	6.3950***	0.0040	

***p < 0.001

### Mapping United States of America (USA) data

**Fig. 4:**
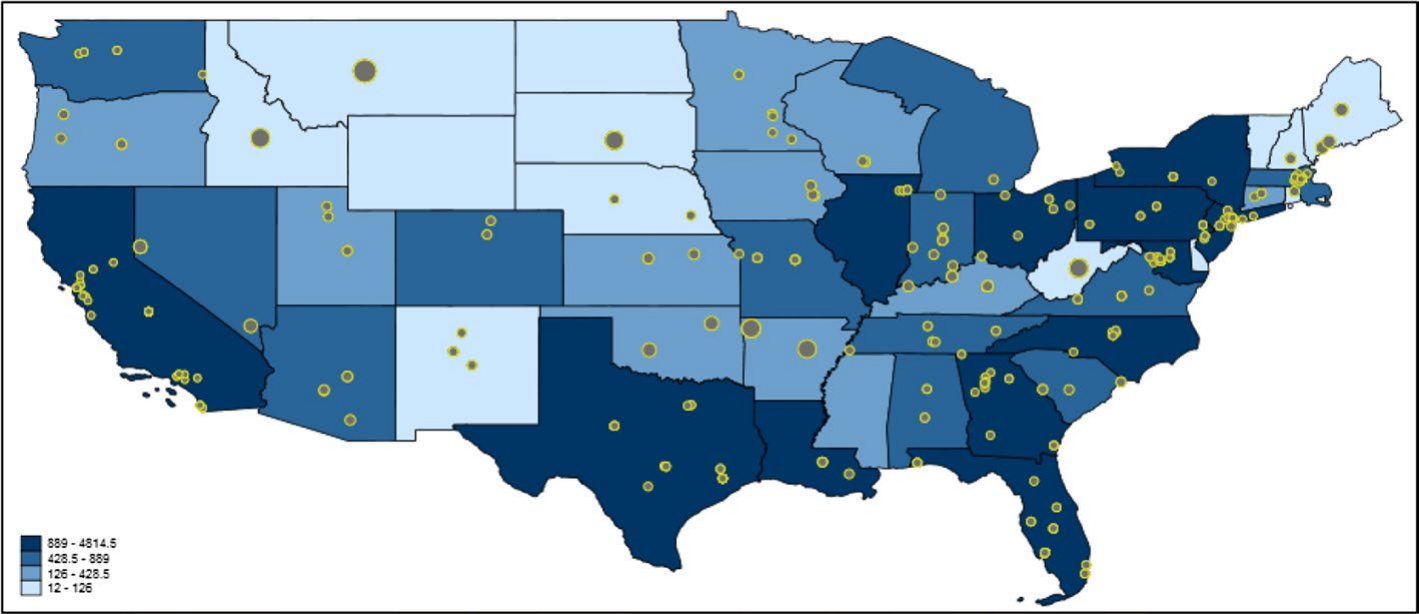
Tweeting on publications dealing with COVID-19/SARS-CoV-2 in the USA. Each tweet is inversely weighted with the number of publications published by authors in the corresponding USA state: the larger the dots, the smaller the research activity. The USA states are colored according to their deaths per one million population (Worldometer’s COVID-19 data 2020)

We did not only use the Twitter data as social-spatial sensors to investigate global trends, but also on a single country. We chose to use the USA as an example because it is one of the most populous nations, has the highest twitter activity and is the most prolific publisher of high-quality science (Garas et al. 2019).^1^ Figure 4 shows publication-based Twitter activity dealing with COVID-19/SARS-CoV-2 in the USA. The blue-colored scheme presents the deaths in the USA states per one million population. The map might show that the deaths in the USA states are in fact related to the number of tweets on COVID-19/SARS-CoV-2 publications. However, there are several USA states with high Twitter activity and high publication output (e.g., the Boston region).

We calculated Poisson regression models with deaths per number of population and number of publications as independent variables and number of tweets as dependent variable. Table 2 reports the results. The results are based on 49 USA states (out of 51, Alaska and Hawaii were excluded) since only USA states with at least one tweet were considered. The percentage changes in expected counts in Table 3 point out that deaths per number of population and Twitter activities are negatively correlated: for a standard deviation increase in the deaths per number of population of a USA state, the expected number of tweets in that state decreases by 10.6 percentage points, holding the USA state’s number of publications constant. The results in Table 3 further show that the influence of the number of publications is significantly greater than that of the deaths per number of population (and positive). In the USA states, there is a strong dependency of Twitter data on the number of publications.

**Table 3:** Coefficients of Poisson regression model with number of tweets as dependent variable (n = 49 USA states)

Independent variable	Coefficient	Standard error	Percentage change in expected count
Deaths per number of population	− 0.0013***	0.0001	− 10.6
Number of publications	0.0744***	0.0005	105.5
Constant	5.1767***	0.0114	

***p < 0.001

**Fig. 5:**
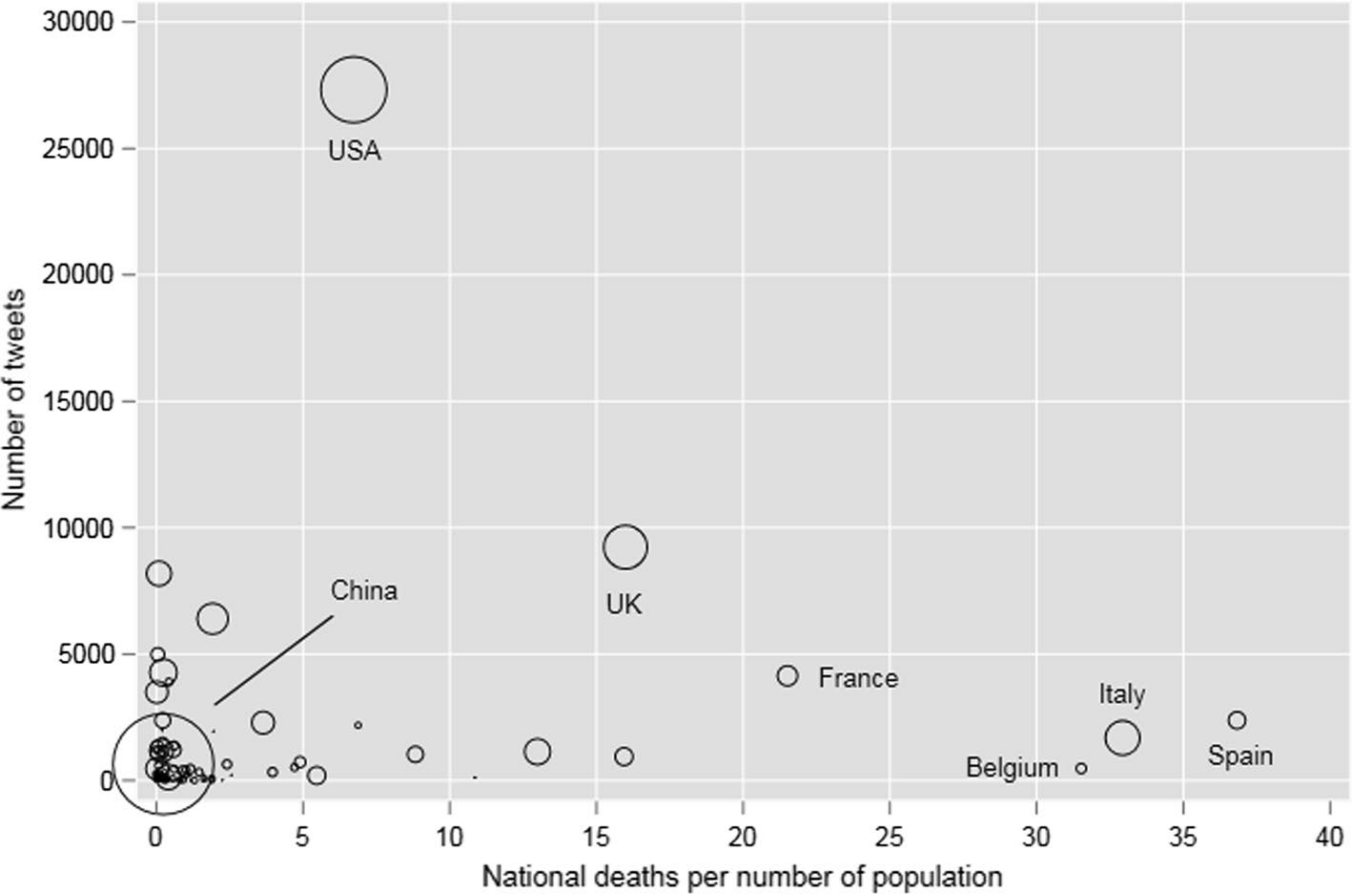
Scatter plot of worldwide data showing deaths per number of population and number of tweets (markers for countries are weighted by the national numbers of publications)

The scatter plot of worldwide data in Fig. 5 demonstrates that at the time of the analysis the USA was an outlier because of lower national deaths per number of population and higher numbers of publications and tweets, when compared to other countries that were significantly impacted by COVID-19/SARS-CoV-2 (e.g., UK, France, Spain, and Italy).

## Discussion

This study demonstrates that Twitter data can be used as social-spatial sensors to monitor research diffusion in a global pandemic using COVID-19/SARS-CoV-2 as an example. Our results suggest that novel research on COVID-19/SARS-CoV-2 publicised through Twitter reaches populations that are concerned about the disease.

Social media can be an effective tool for broadcasting research both within and beyond the academic community (Pulido et al. 2018). Twitter is one of the best social media platforms for sharing scientific research and knowledge because it allows users to post links of recent publications, write a short statement about the research topic and tag keywords with hashtags, so that people who are interested in the research are more likely to see the post (Mandavilli 2011). As well as promoting scientific research, Twitter and other social media platforms can scrutinise research in public, often within hours rather than years, unearthing poor quality inaccurate work (Mandavilli 2011). Governments and research institutions worldwide support a rapid research response to improve understanding of COVID-19/SARS-CoV-2, including the development and testing of therapies and vaccines (Statement from the leaders of the G7 nations 2020).

Our study shows that Twitter can play a vital role in the rapid research response, especially to disseminate research with swift peer review. Our study shows exponential use of Twitter as the intensity of the outbreak has increased. Over 80% of publications extracted from the WHO COIVD-19 database have been cited on Twitter, which is nearly seven times higher than previous studies analysing Twitter data in biomedical sciences (Haustein et al. 2014). Each COVID-19 publication has been tweeted on average 425 times, which is significantly higher than our previous work analyzing Twitter activity of single infectious diseases (on average publications related to Human immunodeficiency virus were tweeted 7 times, tuberculosis were tweeted 8 times, and malaria tweeted 9 times) (Bornmann et al. 2020).

Countries such as China and Iran have blocked Twitter, as well as other social media platforms (Mohammadi et al. 2018). This is reflected in our mapping of worldwide tweet data related to research on COVID-19/SARS-CoV-2 (Fig. 2). The COVID-19/SARS-CoV-2 originated from Wuhan in China’s Hubei province, which quickly became the epicentre for China’s outbreak, followed by a new epicentre in Iran. Both countries have seen a rapid rise in scientific output over the last two decades and their research (Ataie-Ashtiani 2016), coupled with thousands of reported cases, on COVID-19/SARS-CoV-2 has led them to a better understanding of the novel, fast moving virus. However, censorship of social media may have stifled research dissemination and more importantly avoids swift public scrutiny. This may adversely affect the global fight against the disease. Our study suggests that governments should consider relaxing censorship of social media at times of global crisis, such as the COVID-19/SARS-CoV-2 pandemic. Moreover, allowing the public greater access to platforms such as Twitter during a global pandemic can aid the scientific community’s fight against misinformation and pseudoscience (Caulfield 2020).

The USA appears to be an outlier in the worldwide data and the country specific data shows that the USA has a different relationship between tweets and deaths, both of which may be due to the pandemic reaching the USA later than most other countries in the Northern Hemisphere. Another explanation is the difference in geographical clusters of COVID-19/SARS-CoV-2 and research productivity in the USA. On one hand, New York state became the global epicentre of the pandemic after the virus spread through Europe, and over a third of USA COVID-19/SARS-CoV-2 deaths have occurred in New York state, with the majority in New York City (Worldometer’s COVID-19 data 2020). On the other hand, the USA is the most prolific publisher of high-quality science in the world, but the top-performing institutes are concentrated in Massachusetts, California and Maryland (Crew and Jia 2020).

Before concluding, it is important to consider the limitations of this study. This study focusses on Twitter although similar tools exist (e.g., Facebook). We do not expect other results with alternative tools, and the consideration of alternative tools in future studies might be an interesting addition. Although Twitter may be blocked by certain countries, people in these countries may use other microblogging platforms. For example, Sina Weibo is a Chinese microblogging site which is one of their most influential social network platforms, and we cannot determine whether research dissemination has occurred through Sina Weibo or similar platforms.

We have analysed tweets mentioning publications in a quantitative manner which does not account for the association of the tweet with the publication (i.e., a tweet may reference a valid study but claim it to be ‘fake news’ or have another negative overtone). We have not performed any thematic analysis of the tweets in terms of their content (e.g., are tweets referring to testing for COVID-19/SARS-CoV-2, therapies, or vaccines), or quality (e.g., are tweets referring to randomised controlled trials or letters). Since this study is based on publications from the international literature, people who do not know English cannot read the publications. Thus, many people with possible interest in COVID-19/SARS-CoV-2 publications cannot receive the scientific literature and tweets are not expectable. Which of the tweets on COVID-19/SARS-CoV-2 publications are ‘only’ retweets and not original tweets? Would a different handling of retweets yield different results? These are interesting questions which might be an interesting topic of further research.

Despite these limitations, our study has a number of strengths. We have used an evidence-based and robust methodology to clean and analyse data, as well as extracting data from several well-established databases containing real world evidence updated in real time (Bonasio 2014; Global research on coronavirus disease (COVID-19) 2020; Mortality Analyses 2020; Worldometer’s COVID-19 data 2020) Our study comes at a very critical point in time, when a rapid research response is vital to develop therapies and vaccines to slow the COVID-19/SARS-CoV-2 pandemic and lessen the damage of the disease. Our study utilising Twitter data as social-spatial sensors can serve as proof-of-concept for future studies on Twitter and the evolving pandemic.

## Conclusion

COVID-19/SARS-CoV-2 began as a cluster of cases of pneumonia in Wuhan, Hubei Province, but the outbreak quickly progressed from an PHEIC to a pandemic, which highlights the dynamic process of the spread of an infectious disease (Ghebreyesus, 2020; Statement on the second meeting of the International Health Regulations (2005) Emergency Committee regarding the outbreak of novel coronavirus (2019-nCoV) 2020) Our study has simply investigated a snapshot of the relationship between this pandemic, research outputs, and Twitter activity, but demonstrates the importance of how social media platforms can be crucial to spread research with rapid scrutiny, which may also impede the degree of misinformation. We urge governments to pause censorship of social media platforms such as Twitter during these unprecedented times to support the scientific community’s battle against COVID-19/SARS-CoV-2.

## Data Availability

The full data set and the statistical code can be obtained, upon request, from the corresponding author.

## Footnotes

1 https://www.statista.com/statistics/242606/number-of-active-twitter-users-in-selected-countries.
